# 

**Published:** 1847-10

**Authors:** 


### LIBRARY.

Complete Elements of the Science and Art of the Dentist. Translated from the French of M. Desirabode, by *—A—*, ........ .... 401

### JOURNAL.

Article I.—Observations on the Effects of Irritation upon the Deposition and Absorption of Dental Bone. By W. H. Elliot, D. D. S. 3

Article II.—The Teeth, with Respect to the Voice. By A. Hill, D. D. S. .......... 10

Article III.—Extraction of Teeth. By J. Lee, M. D. .......... 23

Article IV.—Observations upon the Importance and Value of a Knowledge of the Collateral Branches of Medicine and Surgery in Connection with Dentistry. By James Robinson, D. D. S., London .......... 29

Article V.—A Course of Lectures on Dental Physiology and Surgery, delivered at the Middlesex Hospital School. By John Tomes, Esq. .......... 33

Article VI.—Letter from Dr. E. Townsend of Philadelphia...... 54

Article VII.—Letter from Dr. Wm. T. G. Morton of Boston...... 56

Article VII.—Inhalation of the Vapor of Sulphuric Ether for the Relief of Pain in Surgical Operations. Dr. Morton's Claims to its Discovery .......... 77

### REVIEW.

Article IX.—A Practical Treatise on the Operation of Surgical and Mechanical Dentistry. By Samuel C. Harbert, Philadelphia, 86

### BIBLIOGRAPHICAL NOTICES.

A Treatise on the Cardinal Points of Dental Science and Practice. By S. M. Shepherd, D. D. S. .......... 93

Advice on the Management of the Teeth. By T. B. Hamlin, D. D. S. 94

The Case Book, or Dentist's Day-Book, Leger, and Recorder, combined. By F. L. Crane, D. D. S. .......... 95

Remarks on the Proper Mode of Administering Sulphuric Ether by Inhalation. By Wm. T. G. Morton, Boston .......... 96

Principles of Human Physiology, with their Chief Applications to Pathology, Hygiene and Forensic Medicine. By Wm. B. Carpenter, M. D., F. R. S., &c. Third American, from the last London edition, with Notes and Additions, by Meredith Clymer, M. D. Philadelphia .......... 96

### MISCELLANEOUS NOTICES.

Eighth Annual Meeting of the American Society of Dental Surgeons, 97

Third Dentition .......... 106

Importance of Medico-dental Education .......... 109

The Work of Desirabode .......... 109

Mr. Tomes' Lectures .......... 109

Tranposition of the Teeth .......... 110

Contributions to the Museum of the Baltimore College of Dent. Surg. 110

Tardiness in the Shedding of the Temporary Teeth .......... 111

To Subscribers in England—Omission—Obituary .......... 112

